# Validity of evaluation scales for post-stroke depression: a systematic review and meta-analysis

**DOI:** 10.1186/s12883-024-03744-7

**Published:** 2024-08-15

**Authors:** Fang Liu, Lei Gong, Huan Zhao, Ying-li Li, Zhiwen Yan, Jun Mu

**Affiliations:** 1 Department of Neurology, The First Affiliated Hospital of Chongqing Medical University, No.1 Youyi Road, Yuzhong District, Chongqing, 400016 China; 2 Department of Neurology, Qingdao Eighth People’s Hospital, Qingdao, Shandong 266000 China

**Keywords:** Post-stroke depression, Depression Scale, Validity, Meta-analysis

## Abstract

**Background:**

Post-stroke depression (PSD) is closely associated with poor stroke prognosis. However, there are some challenges in identifying and assessing PSD. This study aimed to identify scales for PSD diagnosis, assessment, and follow-up that are straightforward, accurate, efficient, and reproducible.

**Methods:**

A systematic literature search was conducted in 7 electronic databases from January 1985 to December 2023.

**Results:**

Thirty-two studies were included, the Patient Health Questionnaire-9 (PHQ-9) and Hamilton Depression Scale (HDRS) had higher diagnostic accuracy for PSD. The sensitivity, specificity, and diagnostic odds ratio of PHQ-9 or diagnosing any depression were 0.82, 0.87, and 29 respectively. And for HDRS, used for diagnosing major depression, the scores were 0.92, 0.89, and 94. Furthermore, these two scales also had higher diagnostic accuracy in assessing depressive symptoms during both the acute and chronic phases of stroke. In patients with post-stroke aphasia and cognitive impairment, highly diagnostic scales have not been identified for assessing depressive symptoms yet.

**Conclusions:**

The PHQ-9 and HDRS scales are recommended to assess PSD. HDRS, which demonstrates high diagnostic performance, can replace structured interviews based on diagnostic criteria.

## Introduction

Stroke is a significant cardiovascular disease, with its incidence rate and associated disease risks being of global concern [1].With the increasing incidence of stroke worldwide, the number of people suffering from post-stroke depression (PSD) has increased significantly [2]. PSD is one of the most common complications after the stroke. The main manifestations are depressive mood and loss of interest, often accompanied by somatic symptoms such as weight loss, insomnia, and fatigue [3, 4]. PSD seriously hinders the recovery of neurological function in stroke patients, leading to prolonged hospital stays loss of social interaction and independent living skills, and even increased stroke recurrence and mortality [5, 6]. Therefore, early diagnosis and treatment of PSD are crucial for prognosis. Currently, the diagnosis of PSD is still based on structured interviews [7]. Since the pathogenesis of PSD is not entirely clear [8], the dual effects of stroke-induced brain damage and mental stress complicate its diagnosis. Presently, PSD is classified as a mental disorder rather than neurological disorder. For example, in the Diagnostic and Statistical Manual of Mental Disorders—5th Edition (DSM-V), PSD is categorized under depressive disorder due to other physical diseases [7]; In the 10th edition of the International Classification of Mental Disorders (ICD-10), it is classified as an organic mental disorder [9]; Similarly, in the Chinese Classification and Diagnostic Standard of Mental Disorders (CCMD-3), it is regarded as a mental disorder caused by cerebrovascular diseases [10]. The diverse diagnostic criteria across to different classification systems further complicate the diagnosis of PSD. Additionally, most of the scales used to assess PSD usually refer to the scales of Major Depressive Disorder (MDD) [4, 11].

There are mainly three types of depression scales. Firstly, self-rating scales, such as Patient Health Questionnaire-9 (PHQ-9), Beck Depression Inventory (BDI), and Self-rating Depression Scale (SDS). Secondly, clinician-rated scales, including Hamilton Depression Rating Scale (HDRS) and Montgomery Asberg Depression Rating Scale (MADRS). Thirdly, depression assessment scales for specific populations are Geriatric Depression Screening Scale (GDS) and Stroke Aphasic Depression Questionnaire (SADQ-10). Due to the lack of uniform standards, clinical studies may apply different scales to assess the same PSD populations or use a single scale to assess PSD populations with different characteristics. The validity of these scales varies widely, leading to differences in the epidemiology, diagnosis, and assessment of PSD. Although some research teams have developed PSD-specific scales, such as Post-Stroke Depression Symptom Inventory (PSDS) [12] and Post-Stroke Depression Prediction Scale (DePreS) [13], their validity is still under clinical evaluation and they are not widely used.

Therefore, it is urgent to identify scales that can simplify the diagnostic process of PSD and facilitate the prognosis evaluation. This meta-analysis aimed to select the accurate, simple and reproducible assessment scales for PSD.

## Methods

### Literature search

Through computer retrieval, seven English electronic databases (PubMed, EMBASE, Medline, Web of Science, Clinical trial.gov, CINAHL, and Cochrane library) were searched for published literature on PSD and scale assessment from January 1985 to December 2023.The search scope included title and abstract, and the language was limited to English. According to the Medical Subject Headings (MeSH), the searched keywords include:

1. Post-stroke depression: ‘post-stroke depression’ or ‘post stroke depression’ or ‘PSD’ or ‘depression after stroke’ or ‘emotional disturbances after stroke’ or ‘emotionalism after stroke’ or ‘vascular depression’ or ‘post stroke depressive disorder’ or ‘depressive disorder after stroke’.
2. Assessment: ‘assessment scale’ or ‘validity’ or ‘measure’ or ‘measures’ or ‘evaluation’.

The retrieval formula was (#1 and #2) not (‘Meta-Analysis’ or ‘Review’ or ‘Systematic Review’).

### Inclusion and exclusion criteria

#### Inclusion criteria were as follows:

1. The studies were original studies, including case-control and cohort studies with a clearly defined period of development or publication.
2. The study content involved the use of depression scales to evaluate PSD
3. Participants met the diagnostic criteria for stroke
4. The evaluation of PSD adhered to the relevant classification and diagnostic criteria (DSM, ICD, CCMD)
5. The study needed to provide the number of patients with stroke and PSD.

#### Exclusion criteria were:

1. Animal studies related to PSD
2. Lack of clear criteria for the diagnosis of stroke
3. Failure to use the diagnostic criteria for PSD based on structured interviews or assessments
4. Researchers did not adopt scientific data collection methods
5. Inappropriate use of statistical methods in research or errors in data analysis
6. Reviews, systematic reviews, dissertations, conference papers, and repeated publications
7. The literature was not in English.

#### Study selection

We included, but not limit to, the following types of scales: ‘The Patient Health Questionnaire-2 (PHQ-2)’, ‘The Patient Health Questionnaire-9 (PHQ-9)’, ‘Center for Epidemiological Studies-Depression(CES-D)’, ‘Montgomery Asberg Depression Rating Scale(MADRS)’, ‘Beck Depression Inventory(BDI)’, ‘Hamilton Depression Rating Scale(HDRS or HAMD)’, ‘Hospital Anxiety and Depression Scale(HADS)”, ‘Self-Rating Depression Scale(SDS)’, ‘The Geriatric Depression Scale(GDS)’, ‘Post stroke depression scale(PSDS)’, ‘ Post Stroke Depression Rating Scale(PSDRS)’, ‘Visual Analog Mood Scale(VAMS)’, and ‘Stroke Aphasic Depression Questionnaire Hospital Version( SADQ-H)’.

### Data extraction

Firstly, the selected studies in the database were entered into the EndNote X9.3.2 software (Thomson Scientific, America). After screening for duplicate studies, the titles and abstracts of the remaining studies were screened again. Secondly, included studies were identified after reading the full text of each study according to the inclusion and exclusion criteria. The extracted data mainly included: author, publication time, number of cases, assessment scales and cut-offs, PSD diagnostic criteria, type of stroke, onset time of stroke when evaluating depressive symptoms, and type of depression.

### Quality evaluation

Two reviewers independently assessed the quality and risk of bias of all included studies using The Risk Of Bias In Non-randomized Studies – of Interventions (ROBINS-I) [14], Any disagreements between the reviewers were be discussed with the superior expert until a consensus was reached.

### Data analysis

The RevMan 5.4 statistical software provided by Cochrane collaboration was used for quality assessment of the data and statistical description. We used Stata15.1 software for meta-analysis and heterogeneity test. In cases where the heterogeneity between studies was *P* > 0.1 and I^2^ < 50%, we employed a fixed-effect model for comprehensive analysis. Conversely, if the heterogeneity between studies was *P* ≤ 0.1 and I^2^ ≥ 50%, the random-effect model was used. We utilized the bivariate mixed-effects model to assess the diagnostic efficacy of the scale, focusing on key evaluation indicators [15] sensitivity, specificity, positive likelihood ratio, negative likelihood ratio, and diagnostic odds ratio. Samples of the scales included in the evaluation must meet the criteria of the bivariate mixed-effects model analysis, with a minimum sample size of 3 (*n* ≥ 3).

Subgroup analysis can be divided into three subgroups: (1) Depression type, which was divided into any depression group and major depression group. Major depression was defined according to the diagnosis of MDD in DSM-V [7]: Patients were required to have five or more of nine depressive symptoms lasting more than two weeks after the stroke event, and at least one of them was 1) mood depression or 2) loss of interest or pleasure. The definition of any depression was broader, according to the depressive disorder definition in DSM-III [16], encompassing adjustment disorder with depressive mood, disorder, and dysthymia. (2) Stroke staging, which was divided into acute phase after stroke (≤ 2 months) and chronic phase after stroke (> 2 months). (3) Specific populations, it includes patients with certain characteristics, such as a comorbid history of pre-stroke depression, stroke with aphasia, cognitive dysfunction, and other features.

## Results

**Fig. 1:**
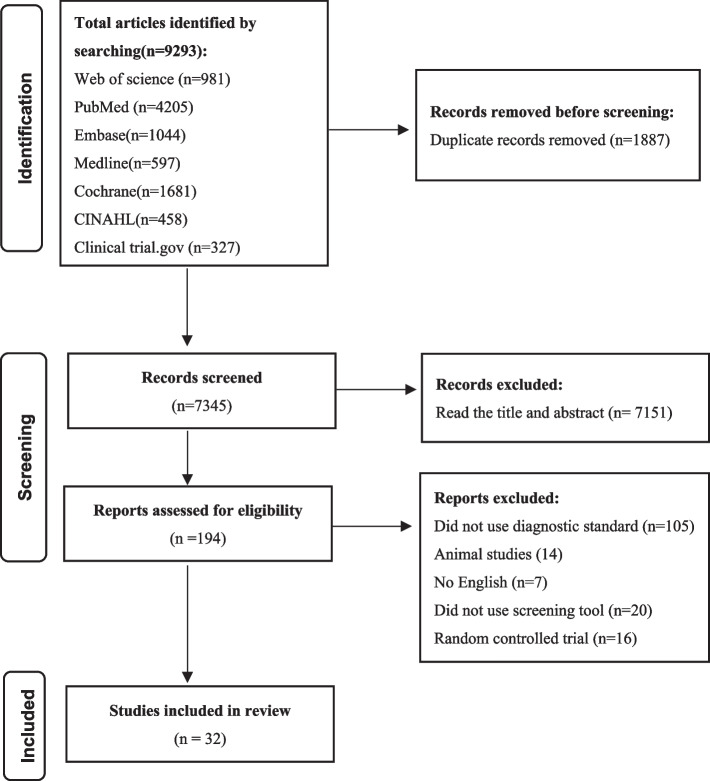
The flow chart of literature screening

**Fig. 2:**
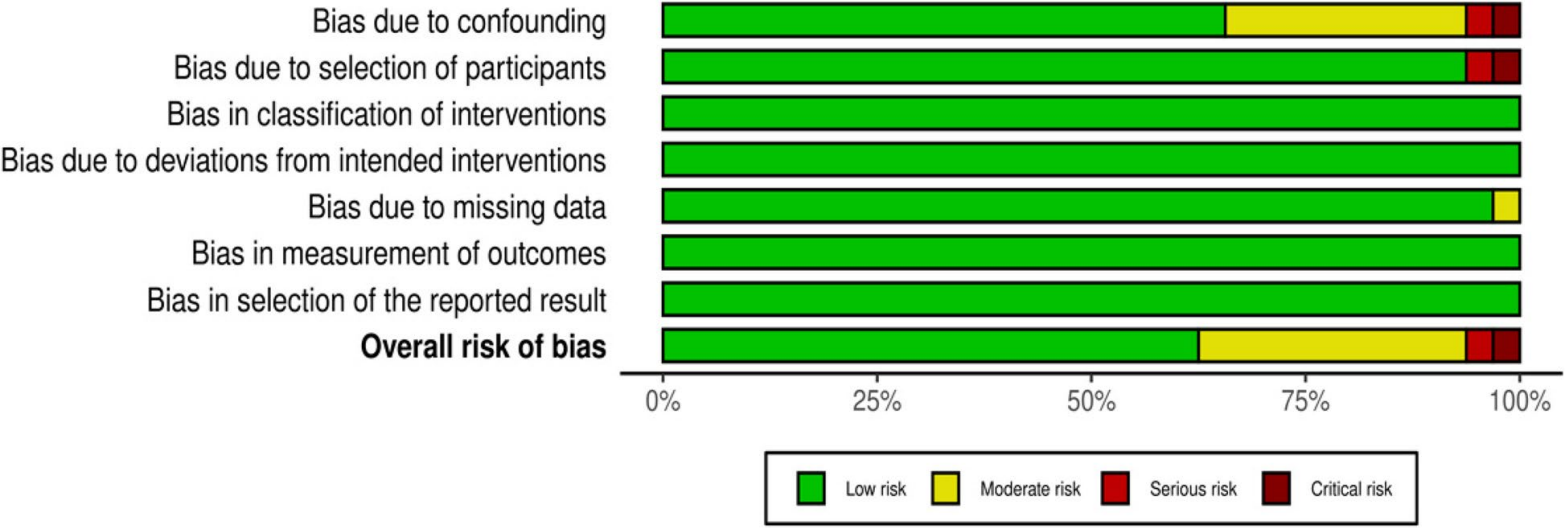
Risk of bias and fitness bar chart

**Fig. 3:**
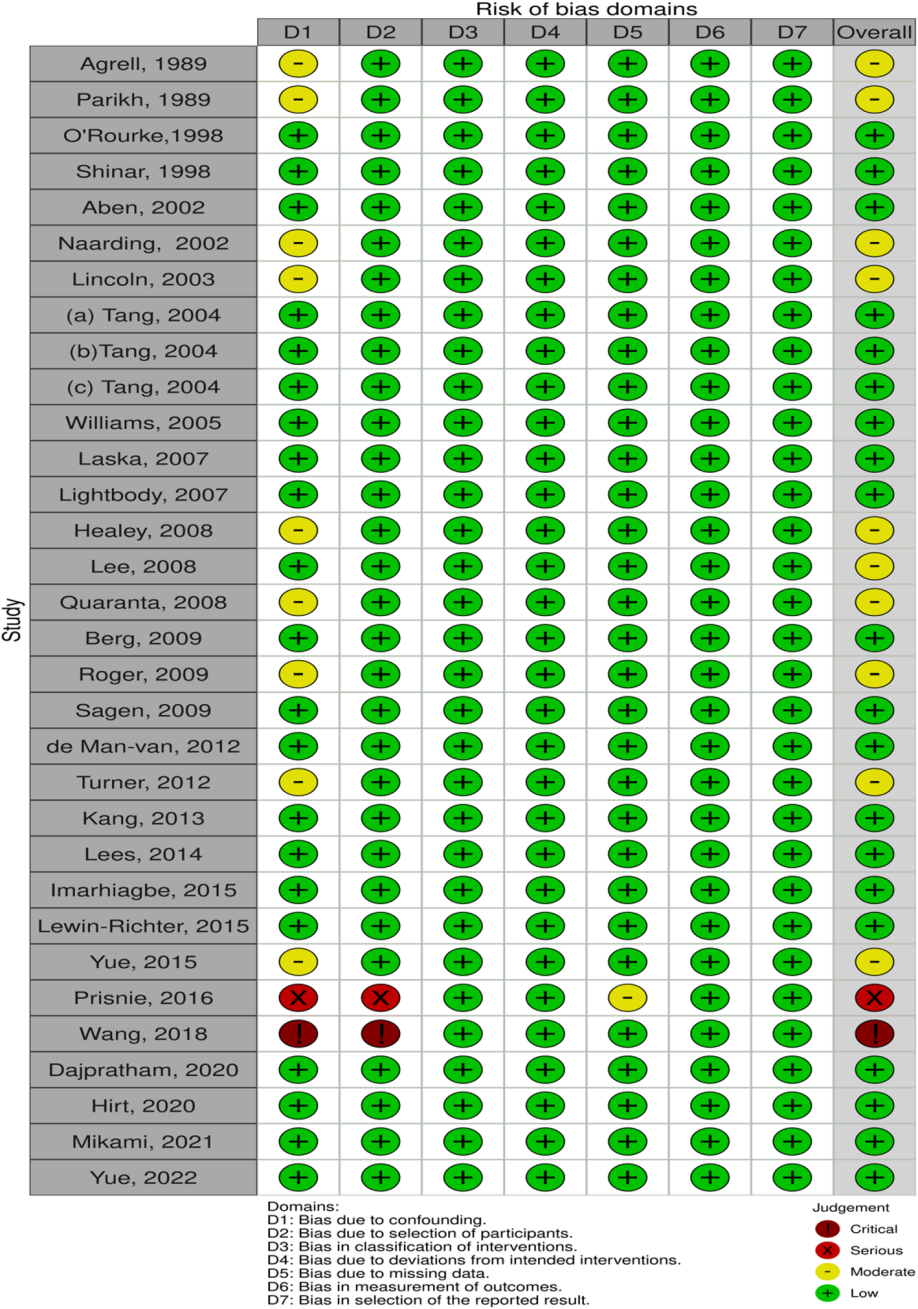
Summary plot of risk of bias and fitness items

This study followed the PRISMA guidelines on reporting [17]. The screening flowchart was shown in Fig. 1.Thirty-two studies [12, 13, 18–47] involving 3865 people aged between 18 and 92 were included. The relevant information from the studies was presented in Table 1. The ROBINS-I was used to evaluate the quality of the included literature. The evaluation results were presented in Fig. 2 and Fig. 3.

**Table 1:** General information about the included literature

**Author**	**Country**	**Study design**	**N**	**Age** **(Mean)**	**Male** **(%)**	**Scale (** **cut-off** **)**	**Diagnostic criteria**	**Stroke type**	**Time**
**Any depression**									
Dajpratham 2020 [19]	Thailand	Cross-sectional	115	64.0	63(54.8)	PHQ-9 (≥6)	DSM-V	Unclear	Post-acute
Imarhiagbe 2015 [23]	Nigeria	Cross-sectional	92	63.8	61(66)	JSSD (≥2.37)	DSM-IV	AIS ICH	0.5-65 months
(a)Yue 2015 [12]	China	Cross-sectional	158	/	114(72.2)	PSDS (≥6) HDRS (≥7)	DSM-IV	AIS ICH	7days-more than 10 years
Lees 2014 [24]	UK	Prospective-longitudinal	69	71.0	41(59)	DISCs (≥2)	MINI (DSM/ICD)	Ischemic ICH	Acute phase
de Man-van 2012 [27]	Netherlands	Prospective	164	70.6	97 (59.1)	PHQ-9 (≥10) PHQ-2 (≥2)	CIDI(DSM-IV/ICD-10)	Ischemic ICH	6-8 weeks
Sagen 2009 [28]	Norway	Prospective	104	64.5	61(58.7)	MADRS (>12)	SCID(DSM-IV)	Ischemic	Unclear
Roger 2009 [29]	USA	Cross-sectional	67	71.0	32(47.8)	GDS-15 (≥3)	SCID(DSM-IV)	AIS	Acute phase
Lee 2008 [32]	China	Cross-sectional	253	/	159(62.8)	GDS-15 (≥15) Smiley Diagrams (sad face)	DSM-IV	Ischemic	1 month
Lightbody 2007 [34]	UK	Cross-sectional	28	/	14(50)	MADRS (≥7)	ICD-10	Unclear	2 weeks
(a)Tang 2004 [37]	China	Cross-sectional	127	75.7	68(53.5)	GDS-15（≥6/7）	DSM-IV	Ischemic ICH	Unclear
(b)Tang 2004 [38]	China	Cross-sectional	100	74.2	55(55)	HADS (≥6/7)	SCID (DSM-III-R)	Ischemic ICH	< 2 weeks
(c)Tang 2004 [39]	China	Cross-sectional	60	71.3	27(45)	HADS (≥3/4)	SCID (DSM-III-R)	Ischemic ICH	< 2 weeks
Lincoln 2003 [40]	UK	Cross-sectional	143	66.0	74(52)	GHQ-28 (≥12)	SCID (DSM-III-R)	Unclear	1month
Aben 2002 [42]	Netherlands		202	68.5	109(55.5)	HADS-D (≥7)	SCID (DSM-IV)	Ischemic	1 month
O'Rourke 1998 [43]	Scotland	Prospective-observational	105	68.0	76(51.7)	GHQ-30 (≥8/9)	DSM-IV	Ischemic	6 months
Agrell 1989 [44]	Sweden	Cross-sectional	40	80.0	18(45)	GDS (≥10) CPRS-D (≥3) ( ZUNG (≥45)	SCID (DSM-III-R)	Unclear	Unclear
Parikh 1988 [45]	USA	Prospective-observational	180	58.4	99(55)	CED-S（≥16）	DSM-III	Ischemic ICH	1 week-1 years
Shinar D 1986 [46]	USA	Cross-sectional	27	56.0	11(40.7)	CED-S（≥16）	DSM-III	Unclear	7 days-6 months
**Major depression**									
Mikami 2021 [18]	Japan	Prospective	48	/	37(77.0)	PHQ-9 (≥9)	DSM-IV	Ischemic	< 6 weeks
Hirt 2020 [13]	Germany	Prospective	93	70.4	57 (61.3)	DePreS (≥0)	CIDI (DSM-IV. ICD-10)	AIS ICH	<1 week
**Major depression**									
Wang 2018 [20]	USA	Cross-sectional	147	69.6	123(83.7)	CES-D (≥10) PHQ-9 (≥10) PHQ-2(≥2) Whooley Questions (≥1)	CDIS (DSM-III)	Unclear	Unclear
Prisnie 2016 [21]	Canada	Cross-sectional	122	60.1	54(44.3)	PHQ-9 (≥13) PHQ-2 (≥3) HDAS-D (≥10) GDS-15(≥7)	SCID: DSM-IV	AIS ICH TIA	Post-acute
Lewin-Richter 2015 [22]	Germany	Prospective-longitudinal	96	66.5	38(39.5)	GDS-15(≥5)	DSM-V	Ischemic	6 months
Turner 2012 [26]	Netherlands	Cross-sectional	72	66.7	38(52.7.)	PHQ-9(>8) PHQ-2(≥3) HADS-D (>5) BDI-II (≥11)	CID(DSM-IV/ICD-10)	Unclear	>3 weeks
Berg 2009 [30]	Finland	Prospective	100	/	68(68.0)	HDRS (≥10) BDI (≥10)	DSM-III-R	Ischemic stroke	2 months 1 year
Quaranta 2008 [31]	Italy	Cross-sectional	143	62.8	81(56.6)	PSDRS (≥9) Ham-D (≥11)	DSM-IV	AIS ICH	Post-acute
Healey 2008 [33]	UK	Cross-sectional	49	78.8	/	BASDEC (≥7) BDI-FS (≥4)	SCID(DSM-IV)	Unclear	16–113 days
Laska 2007 [35]	Sweden	Prospective-observational	89	74.0	50 (56.0)	MADRS (≥10)	DSM-IV	Ischemic ICH	6 months
Naarding 2002 [41]	Netherlands	Cross-sectional	44	70.3	/	HDRS (≥5/6)	DSM-IV	Ischemic ICH TIA	Unclear
Yue 2022 [47]	China	Cross-sectional	170	64.2	105(61.8)	PSDS (≥10) PHQ-9(≥10)	DSM-V	AIS ICH	Unclear
**Any and Major depression**									
Kang 2013 [25]	Korea	Prospective-longitudinal	423	64.5	244(57.7)	MADR (>5/6/8) HDRS (>7/8/12) HADS-D (>5/7) BDI (>8/11)	MINI(DSM-IV)	Ischemic	2 weeks - 1 year
Williams 2005 [36]	Indiana	Cross-sectional	316	/	180(57.0%)	PHQ-9(≥10)	SCID(DSM-IV)	Ischemic stroke	1-2 months

*AIS* Acute ischemic stroke, *ICH* Acute cerebral haemorrhage, *TIA* Transient ischemic attack, *DSM-V* Diagnostic and statistical manual of mental disorders, *ICD-10* International classification of mental disorders, *SCID* Structured clinical interview for DSM, *MINI* Mini-international neuropsychiatric interview, *"Unclear"* the specific type of stroke or onset time in the included population was unknown, "/" No clearly data mentioned in the original study, *CDIS* Computerized version of the national institute of mental health diagnostic interview schedule, *ADRS* Aphasic depression rating scale, *BDI* Beck depression inventory, *CES-D* Center for epidemiological studies-depression, *DePreS* Post-stroke depression prediction scale, *GDS* Geriatric depression screening scale, *HADS* Hospital anxiety and depression scale, *HADS-D* Hospital anxiety and depression scale-depression, *HDRS* Hamilton depression scale, *MADRS* Montgomery asberg depression rating scale, *PHQ-2* The patient health questionnaire-2, *PHQ-9* Patient health questionnaire-9, *PSDRS* Post stroke depression rating scale, *PSDS* Post-stroke depression symptom inventory

### Meta-analysis of scale selection

**Fig. 4:**
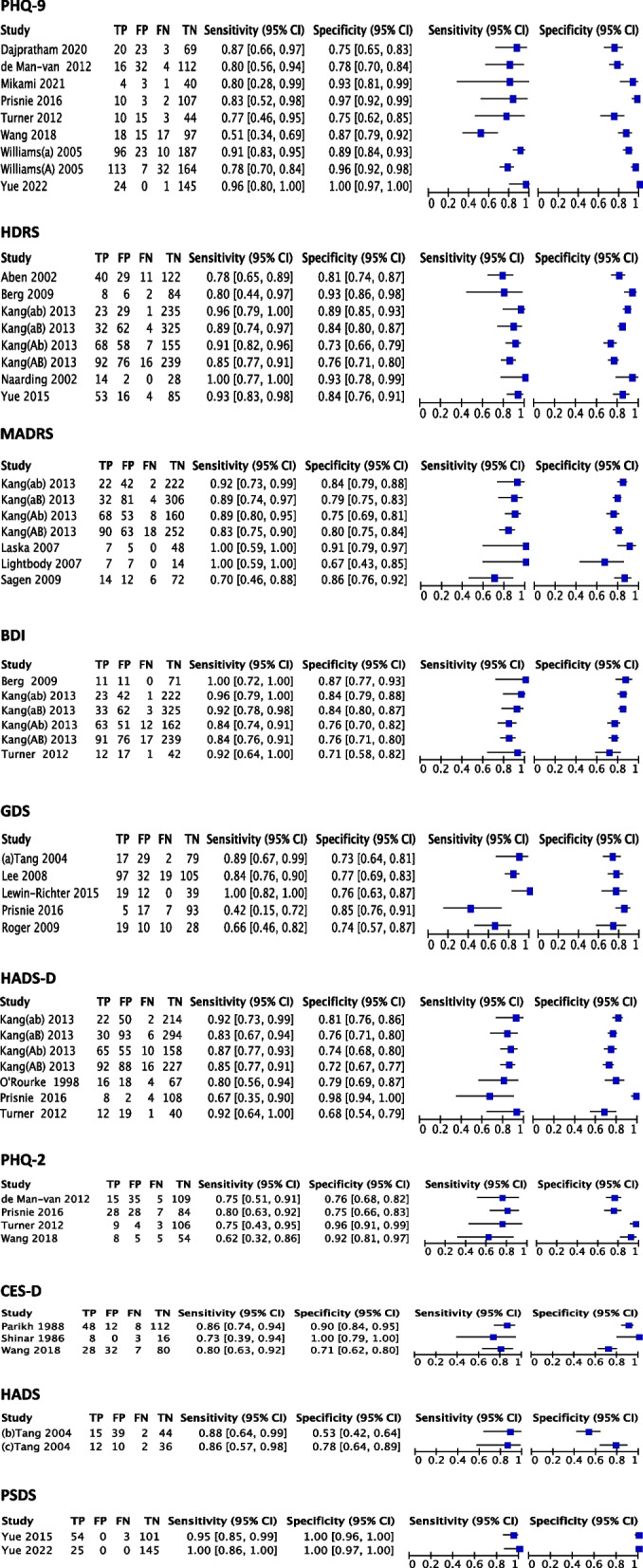
Forest plot of sensitivity and specificity for each scale. PHQ-9: Patient Health Questionnaire-9. HDRS: Hamilton Depression Scale. MADRS: Montgomery Asberg Depression Rating Scale. BDI: Beck Depression Inventory. GDS: Geriatric Depression Screening Scale. HADS-D: Hospital Anxiety and Depression Scale-Depression. PHQ-2: The Patient Health Questionnaire-2. CES-D: Center for Epidemiological Studies-Depression. HADS: Hospital Anxiety and Depression Scale. PSDS: Post-Stroke Depression Symptom Inventory

Sensitivity and specificity of the scales were assessed when the number of articles involved in each scale was two or more (*n* ≥ 2). The study assessed ten scales (PHQ-9, HDRS, MADRS, BDI, GDS, HADS-D, PHQ-2, CES-D, HADS, and PSDS) involving 28 articles. These ten scales had different sensitivities and specificities, and the same scale had different sensitivities and specificities in different studies (Fig. 4).

### Subgroup analysis

#### Depression type

##### Any depression

Five scales were used to assess PSD when depression was classified as any depression in the study. Overall, PHQ-9 had high diagnostic efficacy when both sensitivity and specificity were considered, with a sensitivity of 0.82 (95%CI: 0.72–0.89), specificity 0.87 (95%CI: 0.68–0.95), and diagnostic odds ratio 29 (95%CI: 10.0–84.0); If only higher sensitivity was required, HDRS and MADRS were more advantageous. However, when only higher specificity was considered, PHQ-9 and HADS-D were more advantageous (Table 2).

**Table 2:** Validity analysis of the scale to assess post-stroke depression with any depression

Scale	Number	Sensitivity (95% CI)	Specificity (95% CI)	Positive likelihood ratio (95% CI)	Negative likelihood Ratio (95% CI)	Diagnostic odds Ratio (95% CI)
PHQ-9	3	0.82(0.72, 0.89)	0.87(0.68, 0.95)	6.1(2.4, 15.5)	0.21(0.14, 0.33)	29(10.0, 84.0)
MADRS	4	0.85(0.75, 0.92)	0.79(0.73, 0.84)	4.0(3.3, 4.9)	0.19(0.11, 0.31)	21(13.0, 36.0)
HDRS	4	0.87(0.81, 0.91)	0.77(0.73, 0.81)	3.8(3.2, 4.6)	0.17(0.12, 0.25)	23(14.0, 36.0)
HADS-D	4	0.81(0.70, 0.89)	0.85(0.65, 0.95)	5.4(2.2, 13.0)	0.22(0.15, 0.33)	25(11.0, 57.0)
GDS	4	0.74(0.54, 0.88)	0.78(0.72, 0.83)	3.4(2.70, 4.3)	0.33(0.18, 0.62)	10(5.0, 22.0)

*PHQ-9* Patient health questionnaire-9, *MADRS* Montgomery asberg depression rating scale, *HDRS* Hamilton depression scale, *HADS-D* Hospital anxiety and depression scale-depression, *GDS* Geriatric depression screening scale

##### Major depression

When classifying depression as major depression, six scales were used to assess PSD. Overall, when the sensitivity and specificity were considered together, HDRS had a high diagnostic power, with a sensitivity of 0.92 (95%CI: 0.82–0.97), specificity of 0.89 (95%CI: 0.84–0.92), and diagnostic odds ratio of 94 (95%CI: 32–281); Likewise, if only the sensitivity was considered, BDI, HDRS, MADRS had the advantage; but for higher specificity, PHQ-9 and PHQ-2 had the advantage (Table 3).

**Table 3:** Validity analysis of the scale to assess post-stroke depression with major depression

Scale	Number	Sensitivity (95% CI)	Specificity (95% CI)	Positive likelihood ratio (95% CI)	Negative likelihood Ratio (95% CI)	Diagnostic odds Ratio (95% CI)
PHQ-9	6	0.84(0.67, 0.94)	0.94(0.82, 0.98)	13.9(4.1, 46.7)	0.17(0.07, 0.40)	83(12, 564)
PHQ-2	3	0.73(0.58, 0.84)	0.90(0.76, 0.96)	7.5(3.0, 19.0)	0.30(0.19, 0.47)	25(9, 73)
MADRS	3	0.92(0.79, 0.97)	0.83(0.77, 0.87)	5.4(3.8, 7.6)	0.10(0.03, 0.27)	56(16, 200)
HDRS	4	0.92(0.82, 0.97)	0.89(0.84, 0.92)	8.2(5.5, 12.2)	0.09(0.04, 0.21)	94(32, 281)
HADS-D	3	0.88(0.78, 0.94)	0.77(0.73, 0.81)	3.8(3.1, 4.7)	0.16(0.08, 0.31)	24(11, 54)
BDI	4	0.94(0.86, 0.98)	0.83(0.81, 0.86)	5.6(4.8, 6.7)	0.07(0.03, 0.17)	79(31, 199)

*PHQ-9* Patient health questionnaire-9, *PHQ-2* The patient health questionnaire-2, *MADRS* Montgomery asberg depression rating scale, *HDRS* Hamilton depression scale, *HADS-D* Hospital anxiety and depression scale-depression, *BDI* Beck depression inventory

#### Staging of stroke

##### Acute phase after stroke

A total of three scales were used to assess PSD in the acute phase of stroke. PHQ-9 had high diagnostic performance when both sensitivity and specificity were considered, with a sensitivity of 0.85 (95%CI: 0.78–0.91), specificity of 0.90 (95%CI: 0.82–0.95), diagnostic odds ratio of 55 (95%CI: 30–102); If only higher sensitivity was considered, MADRS was more favorable, and if only higher specificity was considered, PHQ-9 was more favorable (Table 4).

**Table 4:** Validity analysis of the scale to assess post-stroke depression in the acute phase of stroke

Scale	Number	Sensitivity (95% CI)	Specificity (95% CI)	Positive likelihood ratio (95% CI)	Negative likelihood Ratio (95% CI)	Diagnostic odds Ratio (95% CI)
PHQ-9	4	0.85(0.78, 0.91)	0.90(0.82, 0.95)	9.0(4.7, 17.1)	0.16(0.11, 0.24)	55(30, 102)
MADRS	4	0.86(0.80, 0.91)	0.80(0.77, 0.83)	4.3(3.7, 5.0)	0.17(0.12, 0.26)	25(15, 40)
HDRS	4	0.83(0.76, 0.89)	0.84(0.76, 0.89)	5.1(3.5, 7.5)	0.20(0.13, 0.29)	26(14, 47)

*PHQ-9* Patient health questionnaire-9, *MADRS* Montgomery asberg depression rating scale, *HDRS* Hamilton depression scale

##### Chronic phase after stroke

There were eight scales to assess PSD in the chronic phase of stroke. Overall, when high sensitivity and specificity were considered together, HDRS had high diagnostic power, with a sensitivity of 0.94 (95%CI: 0.87–0.98), specificity of 0.85 (95%CI: 0.76–0.91), diagnostic odds ratio of 96 (95%CI: 27–346); If only higher sensitivity was considered, HDRS and BDI had the advantage, on the contrary, if only higher specificity was considered, PHQ-2 and CES-D had the advantage (Table 5).

**Table5:** Validity analysis of the scale to assess post-stroke depression in the chronic phase of stroke

Scale	Number	Sensitivity (95% CI)	Specificity (95% CI)	Positive likelihood ratio (95% CI)	Negative likelihood Ratio (95% CI)	Diagnostic odds Ratio (95% CI)
PHQ-9	4	0.75(0.56, 0.88)	0.86(0.72, 0.94)	5.5(2.4, 12.5)	0.29(0.15, 0.56)	19(6, 65)
PHQ-2	3	0.73(0.58, 0.84)	0.90(0.76, 0.96)	7.5(3.0, 19.0)	0.30(0.19, 0.47)	25(9, 73)
MADRS	3	0.85(0.74, 0.92)	0.82(0.76, 0.87)	4.7(3.6, 6.2)	0.18(0.11, 0.32)	26(14, 147)
HDRS	4	0.94(0.87, 0.98)	0.85(0.76, 0.91)	6.3(3.7, 10.6)	0.07(0.03, 0.16)	96(27, 346)
HADS-D	5	0.84(0.74, 0.91)	0.84(0.68, 0.93)	5.1(2.6, 10.2)	0.19(0.12, 0.30)	27(14, 54)
GDS	3	0.87(0.41, 0.99)	0.78(0.70, 0.84)	3.9(3.0, 5.2)	0.16(0.02, 1.13)	25(3, 193)
CES-D	3	0.82(0.74, 0.89)	0.88(0.66, 0.97)	7.0(2.2, 22.*6*)	0.20(0.12, 0.32)	35(8, 149)
BDI	4	0.92(0.78, 0.98)	0.81(0.75, 0.85)	4.8(3.4, 6.6)	0.09(0.03, 0.31)	51(12, 215)

*PHQ-9* Patient health questionnaire-9, *PHQ-2* The patient health questionnaire-2, *MADRS* Montgomery asberg depression rating scale, *HDRS* Hamilton depression scale, *HADS-D* Hospital anxiety and depression scale-depression, *GDS* Geriatric depression screening scale, *CES-D* Center for epidemiological studies-depression, *BDI* Beck depression inventory

#### Specific populations

For analysis the specific populations for PSD, 9 out of 32 studies compared the baseline data characteristics of depressed and nondepressed patients after stroke. According to the previous and included data in this study, a total of seven specific populations were analyzed, with clinical features including cognitive impairment, severe aphasia, pre-onset antidepressant medication, first stroke, severity of neurological deficit, educational level, and previous psychiatric history (Table 6). However, due to the different inclusion and exclusion criteria and priorities among the original studies, the included data were insufficient, and effective statistical analysis could not be performed.

**Table 6:** Scale selection for specific populations of post-stroke depression

Author	Scale	MMSE (SD)	Severe aphasia	Antidepressant (%)	First stroke	NIHSS (SD)	Education level (%)	Psychiatric history (%)
Mikami 2021 [18]	PHQ-9	29.6(0.5)	Exclude	3(60.0)	/	4.0(5.6)	/	3(60.0)
Dajpratham 2020 [19]	PHQ-9	/	Exclude	/	Yes	/	10(43.5)	Exclude
Wang 2018 [20]	CES-D, PHQ-9, PHQ-2 Whooley Questions	/	/	15 (43.0)	/	/	30 (86.0)	/
Prisnie 2016 [21]	PHQ-9, PHQ-2, HDAS-D, GDS-15	/	Exclude	5(41.7)	NO	/	4(33.3)	//
(b)Yue 2015 [48]	PSDS, HDRS	/	/	Exclude	/	/	/	Exclude
Yue 2022 [47]	PSDS, PHQ-9	/	/	Exclude	/	/	/	Exclude
Imarhiagbe 2015 [23]	JSS-D	/	Exclude	/	NO	/	/	/
de Man-van 2012 [27]	PHQ-9 PHQ-2	/	/	/	/	/	/	/
Roger 2009 [29]	GDS-15	/	Exclude	/	/	/	/	/

The data are all data of patients diagnosed with post-stroke depression, *"/"* not mentioned in the original study, *"Exclude"* the original study has been excluded, *"Yes"* the patients included in the original study are all first-time stroke patients, *"NO"* Not all patients included in the original study were first-time stroke patients, *Educational level* high school level or above, *MMSE* Mini-mental state examination, *NIHSS* National institutes of health stroke scale, *PHQ-9* Patient health questionnaire-9, *CES-D* Center for epidemiological studies-depression, P*HQ-2* The patient health questionnaire-2, *HADS-D* Hospital anxiety and depression scale-depression, *GDS-15* Geriatric depression screening scale-15, *PSDS* Post-stroke depression symptom inventory, *JSS-D* Japan stroke scale-depression scale

#### Prevalence of PSD

**Fig. 5:**
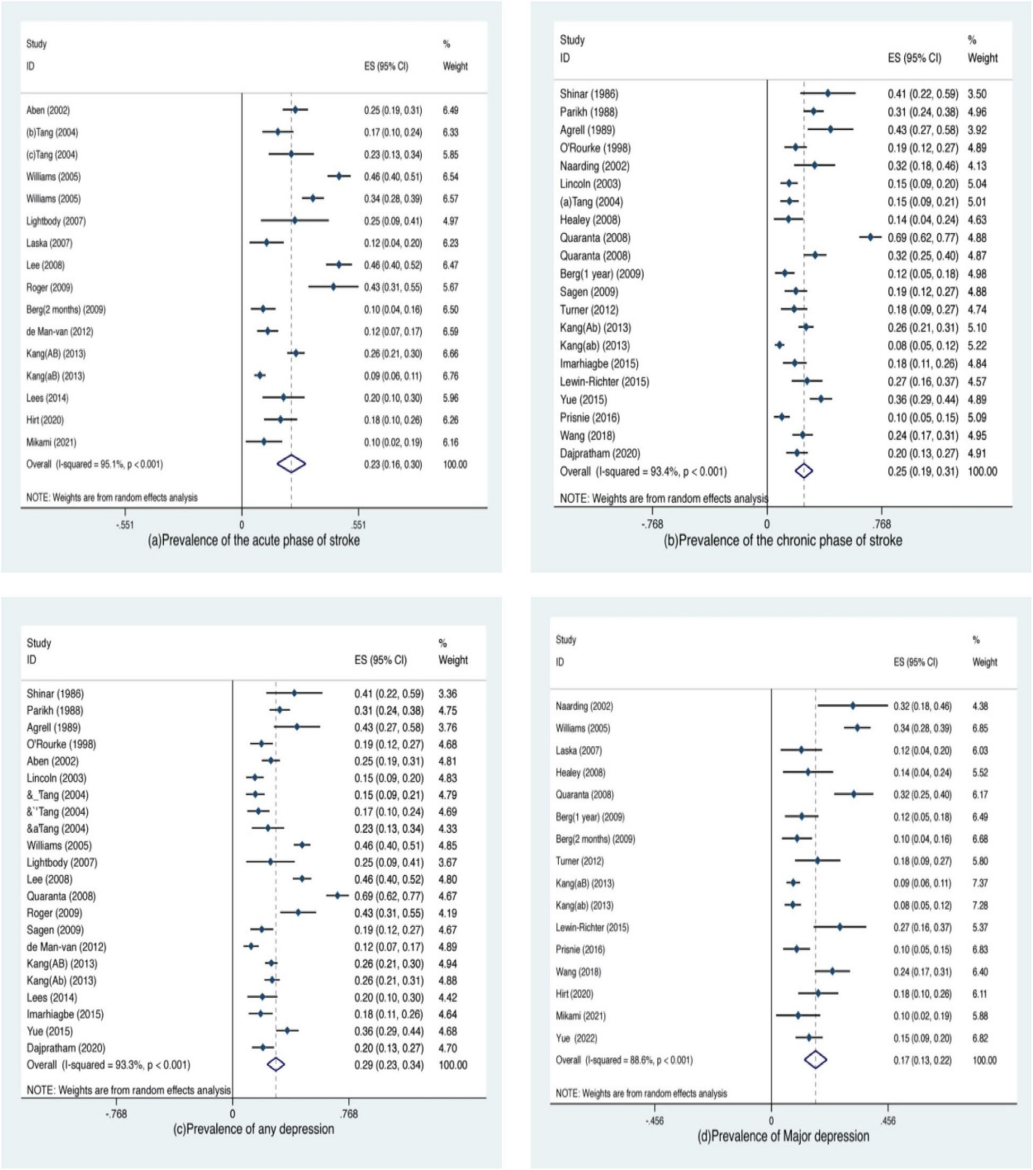
Prevalence of post-stroke depression in different stroke periods and depression types (forest plots)

The results showed that the prevalence of PSD was approximately 17.0% to 29.0%, and the prevalence of PSD in the acute and chronic phases of stroke was 0.23 (95%CI 0.16–0.32) and 0.25 (95%CI 0.19–0.31), respectively. The prevalence of PSD for any depression and major depression was 0.29 (95%CI 0.23–0.34) and 0.17 (95%CI 0.13–0.22), respectively (Table 7 and Fig. 5).

**Table 7:** Prevalence of post-stroke depression in different stroke periods and depression types

Classification	Group	Prevalence (95%CI)
Staging of stroke	Acute phase	0.23(0.16, 0.32)
	Chronic phase	0.25(0.19, 0.31)
Depression type	Any depression	0.29(0.23, 0.34)
	Major depression	0.17(0.13, 0.22)

## Discussion

Thirty-two studies were analyzed to determine the best assessment scale for PSD. The results showed that each of these scales (PHQ-9, HDRS, MADRS, BDI, PHQ-2, CES-D, and HADS-D) had different degrees of advantage in diagnosing PSD based on depression type and stroke staging. When evaluating PSD, PHQ-9 exhibits higher diagnostic efficacy for any depression and acute phase after stroke compared to other scales. Conversely, HDRS performs better for major depression and chronic phase after stroke. Due to limitations in the data included in the literature, no effective scale has been found yet to accurately assess PSD patients with combined aphasia and cognitive impairments.

Currently, many studies utilize depression assessment scales for diagnosing PSD. However, controversy remains, as some studies suggest that these scales are not suitable for diagnosing PSD but rather for assessing the severity of depressive symptoms, treatment efficacy, or prognosis [48, 49]. Whether a scale can substitute for structured interviews in diagnosing PSD depends on its diagnostic accuracy. Our analysis revealed that PHQ-9 and HDRS performed excellently in identifying depressive symptoms and severity. The PHQ-9 is a self-rating scale consisting of 9 items with high sensitivity and specificity [50, 51]. It has been widely used in screening of PSD, because of its simplicity, less time-consuming, and low requirements for patient cooperation. HDRS, introduced in 1960, comprises seven categories, including items for somatic symptoms [52]. It is well known that in the chronic phase of stroke, many patients experience atypical depressive symptoms, such as gastrointestinal symptoms, weight loss, general pain, fatigue, and other physical discomforts [53]. HDRS can be used to assess these patients more accurately. Additionally, studies have shown that HDRS is not only uesd to evaluate the severity of PSD, but also to assess the efficacy of antidepressant treatment [54, 55].

Burton conducted a review of the scales used for screening post-stroke mood disorders in 2015 [56]. They focus on mood disorders after stoke, which include various emotions, such as major depression, any degree of depression, or anxiety. Meader also conducted a related meta-analysis in 2014, which included 24 studies involving 2907 patients [57], the results showed that many scales could screen the PSD, such as CESD, HDRS, and PHQ-9. However, these scales should not be used alone but should be combined with detailed clinical assessments. In comparison to Burton’s and Meader's studies, our study included thirty-two studies, and we provided a clearer description of the stage of stroke and the type of depression for PSD. Additionally, we discussed the selection of scales for PSD in special populations and analyzed the prevalence of PSD.

For the staging of stroke, there is still no unified conclusion at present, and the duration of stroke will affect the symptoms of PSD [58, 59]. Some studies recommend assessing PSD at 2 or 8 weeks after stroke, and Toso 's study found that PSD most occurred within 3 months after stroke [60]. In our study, stroke was staged into the acute phase (within 2 months of stroke onset) and chronic phase (2 months after stroke onset). According to the severity of depression, Robinson classified PSD into mild PSD (mild depression) and severe PSD (severe depression). Mild PSD corresponds to dysthymia in DSM-III, while severe PSD meets the diagnostic criteria for MDD [61]. Therefore, in this study, PSD was divided into two groups: any depression and major depression, and it should be emphasized that any depression included major depression and mild depression.

This study aimed to analyze which scale was more effective in identifying and assessing depressive symptoms in the specific population with PSD. However, due to the different inclusion and exclusion criteria and priorities among the original studies, the included data were insufficient, and effective statistical analysis could not be performed. Stroke patients often experience complications such as aphasia and cognitive dysfunction, which can exacerbate PSD. A related study found that post-stroke aphasia patients are more likely to suffer from depression than non-aphasia patients [62]. According to a systematic review by Mariska, there was insufficient evidence supporting the use of a specific scale to evaluate the depressive symptoms in aphasia patients, and the evidence level of existing studies was relatively low [63]. In addition, relevant studies have shown that post-stroke cognitive impairment (PSCI) was closely related to the occurrence of PSD [64, 65]. Impairment oognitive function can affect the evaluation of depressive symptoms to varying degrees. At present, cognitive function scales based on the assessment of Alzheimer's disease are often used in clinical work to assess PSCI, such as Mini-Mental State Examination (MMSE), Montreal Cognitive Assessment Scale (MoCA), and Cambridge Geriatric Cognitive Scale (CAMCOG). However, the organic damage of cerebral parenchyma in stroke patients, along with complications such as aphasia, visual impairment, dyslexia, and limb dysfunction, can impose limitations in the evaluation of PSCI using the aforementioned scales [66, 67]. Hence, further research is warranted to determine the most suitable scales for assessing depressive symptoms in patients with post-stroke aphasia and cognitive impairment.

The results of the study revealed that the prevalence of PSD, determined through standard structured interviews, ranged from 17.0% to 29.0%. Previous studies by Ayerbe and Hackett indicated that approximately one-third of stroke patients experienced varying degrees of depression within five years after the stroke event [68–70]. It is important to note that the assessment of prevalence was primarily conducted using depression scales. Many factors affect the prevalence of the PSD, such as the population, time, and place of assessment. Nowadays, there is a divergence of opinions regarding whether the timing of PSD assessment influences the prevalence of depression. Some studies showed that the prevalence of depression in the acute phase after stroke was higher than in the chronic phase, and the prevalence gradually decreases over time [71–73], However, another study found no difference in the prevalence of PSD in the early, middle, and late stages of stroke [74]. Therefore, more high-quality prospective studies will be needed in the future to clarify this issue.

## Limitations

There are also some limitations in this study [1]. This study was a secondary analysis, and the included studies exhibited significant heterogeneity due to variations in diagnostic thresholds for each scale. Additionally, the optimal diagnostic cut-off of each scale was not analyzed, so it needs to clarify in future studies [2]. Data limitations and mismatches between the original studies hindered subgroup analyses of scale selection, thereby preventing adequate analyses for different types and severity of stroke, aphasia population, the elderly population, individuals with a history of depression, and other populations. In the future, developing more comprehensive research protocols for PSD is crucial.

## Conclusion

In conclusion, there are various scales to evaluate PSD. To improve diagnostic effectiveness, a variety of scales can be used for dynamic, multi-directional evaluation and follow-up. The PHQ-9 and HDRS are recommended for the evaluation PSD due to their high diagnostic efficiency. Structured interviews based on diagnostic criteria can determine whether stroke patients have depressive symptoms, and depression scales can further determine the severity of symptoms. It is recommended to replace the structured interviews based on diagnostic criteria with rating scales, such as HDRS, with high diagnostic efficacy. Currently, there is still a lack of depression scales for evaluating patients with post-stroke aphasia and cognitive dysfunction.

## Data Availability

No datasets were generated or analysed during the current study.

## Abbreviations

ADRS: Aphasic Depression Rating Scale
BDI: Beck Depression Inventory
CAMCOG: Cambridge Geriatric Cognitive Scale
CCMD-3: Chinese Classification and Diagnostic Standard of Mental Disorders
CES-D: Center for Epidemiological Studies-Depression
CGI-S: Clinical Global Impression-Scale
DePreS: Post-Stroke Depression Prediction Scale
DSM-V: Diagnostic and Statistical Manual of Mental Disorders
GDS: Geriatric Depression Screening Scale
HADS: Hospital Anxiety and Depression Scale
HADS-D: Hospital Anxiety and Depression Scale—Depression
HDRS: Hamilton Depression Scale
ICD-10: International Classification of Mental Disorders
MADRS: Montgomery Asberg Depression Rating Scale
MDD: Major Depressive Disorder
MeSH: Medical Subject Headings
MMSE: Mini-Mental State Examination
MoCA: Montreal Cognitive Assessment Scale
PHQ-2: The Patient Health Questionnaire-2
PHQ-9: Patient Health Questionnaire-9
PSCI: Post-Stroke Cognitive Impairment
PSD: Post-stroke depression
PSDRS: Post Stroke Depression Rating Scale
PSDS: Post-Stroke Depression Symptom Inventory
SADQ-10: Stroke Aphasic Depression Questionnaire
SDS: Self-rating Depression Scale
SODS: Signs of Depression Scale
VAMS: Visual Analog Mood Scale
VASES: Visual Analogue self-esteem Scale
